# Association between grip strength and albuminuria in the general United States population: NHANES 2011–2014

**DOI:** 10.3389/fpubh.2024.1353881

**Published:** 2024-04-19

**Authors:** Laisha Yan, Xiaoyan Hu, Shanshan Wu, Lina Chen, Shunying Zhao

**Affiliations:** Department of Cardiosurgery Intensive Care Unit, Ningbo Medical Centre Li Huili Hospital, Ningbo, China

**Keywords:** grip strength, albuminuria, sarcopenia, NHANES, muscle strength

## Abstract

**Background:**

Grip strength has been shown to be associated with chronic renal insufficiency, but the relationship between grip strength and albuminuria has not been confirmed. In this study, we used NHANES data to explore the association between grip strength and albuminuria in a US population.

**Methods:**

In this analytical study, we utilized data sourced from the National Health and Nutrition Examination Survey (NHANES), specifically spanning the years 2011 to 2014. The dataset included 9,638 participants aged 20 years or older. After adjusting for potential confounders, multiple regression models were developed to infer the interrelationship between grip strength and albumin to creatinine ratio (ACR), and subgroup analyses were conducted.

**Results:**

After adjusting for all covariates, ACR by 0.49 mg/g [−0.49 (95% CI: −0.93, −0.04)] for each 1 kg increase in grip strength decreased. Subgroup analysis showed that gender, age, hyperlipidemia, hypertension, diabetes mellitus, smoking, alcohol consumption and body mass index did not influence the negative correlation between grip strength and albuminuria.

**Conclusion:**

There is a negative correlation between grip strength and albuminuria in the general U.S. population.

## Introduction

Sarcopenia, defined by the accelerated decline in skeletal muscle mass and functionality, represents a progressive and widespread condition. This disorder is associated with various adverse health consequences, including an elevated risk of falls, declining functional abilities, weakness, and a higher likelihood of mortality (1). Diagnosing sarcopenia involves evaluating both muscle mass and muscle function, with grip strength being the simplest clinical measure of the latter (2). Grip strength declines progressively after middle age and has been shown to be associated with a variety of diseases such as cognitive performance, risk of depression, bone density, vascular calcification, diabetes, and cancer in the middle-aged and older population (3–8).

Previous studies have established a significant correlation between renal insufficiency and lower muscle strength (8, 9). Grip strength is not only an independent predictor of renal outcomes in patients with chronic kidney disease (CKD) but also a critical prognostic indicator in this demographic (10–13). For instance, pre-transplant grip strength has been identified as the strongest predictor of post-kidney transplantation outcomes (14). Additionally, the correlation between grip strength and estimated glomerular filtration rate (eGFR) has been consistently observed across genders (9). These studies primarily focus on the assessment of nutrition and prognosis in CKD patients through grip strength evaluation.

Albuminuria is a well-known marker of kidney disease and occurs very commonly in the population (15). Recent research has not only reaffirmed its role as a marker of renal injury but also highlighted its predictive value for outcomes in CKD patients. Furthermore, albuminuria is strongly linked with an array of cardiovascular disease risks and prognoses (16–18). Although the underlying mechanisms remain to be fully elucidated, albuminuria contributes to progressive renal and cardiac damage. This suggests that albuminuria not only serves as a marker for renal and cardiovascular risks but also represents a target for protective interventions (19). The primary objective of our study is to explore the independent association between grip strength and albuminuria in the general population.

## Methods

### Study population

**Figure 1:**
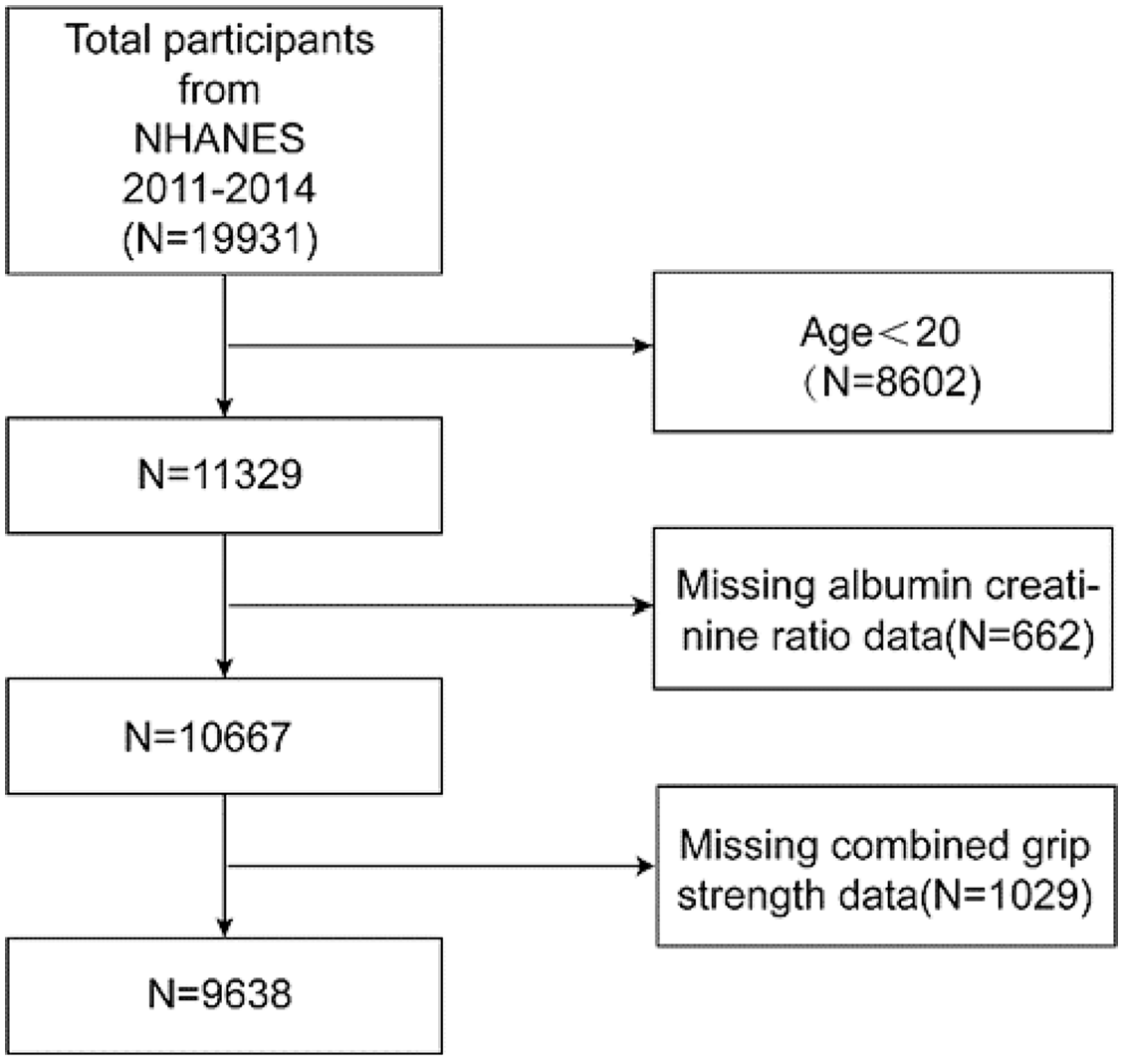
Flowchart of participant selection.

The National Health and Nutrition Examination Survey (NHANES), a crucial initiative of the Centers for Disease Control and Prevention (CDC) in the United States, is structured to evaluate the health and nutritional conditions of both adults and children nationwide. Our study incorporated data from two NHANES cycles, spanning 2011–2014, with a total of 19,931 participants. After excluding individuals under the age of 20, those with missing albumin to creatinine ratio (ACR) data, and participants with incomplete grip strength information, the final cohort consisted of 9,638 subjects. The inclusion and exclusion processes are depicted in Figure 1.

### Study variables

The independent variable of the study was grip strength, which in this paper is defined as the composite grip strength measured by gripometry, excluding subjects with arm or hand impairments, missing thumbs, paralysis, or those who had hand or wrist surgery within the past three months. For each hand, three tests were conducted, alternating between each test, with a pause of 60 s between measurements on the same hand. The overall grip strength was determined by summing the highest readings from each hand.

The dependent variable was ACR, and the researchers used solid-phase fluorescence immunoassay and the Jaffe rate method to measure protein and creatinine, respectively, in random urine samples. Random urine albumin (mg/mL) and urinary creatinine (mg/dL) were converted to ACR in mg/g.

The selection of covariates was based on the results of previous studies and clinical expertise. They included the following demographic, health, and biochemical indicators: age, sex, race, education, alcohol consumption, smoking, hypertension, hyperlipidemia, diabetes mellitus, total cholesterol (TC), high-density lipoprotein cholesterol (HDL-C), low-density lipoprotein cholesterol (LDL-C), triglycerides (TG), body mass index (BMI), creatinine (Cr), Hemoglobin A1c (HbA1c), eGFR, and uric acid (UA). The history of hypertension and hyperlipidemia was obtained through questionnaire. Diabetes was diagnosed based on one or more criteria: obtained through questionnaire, fasting plasma glucose (FPG) ≥ 7.0 mmol/L, random blood glucose ≥11.1 mmol/L, HbA1c > 6.5%, or a 2-h oral glucose tolerance test (OGTT) with blood glucose levels ≥11.1 mmol/L.BMI was classified into three categories: less than 25 kg/m2 for normal weight, 25–29.9 kg/m2 for overweight, and 30 kg/m2 or more for obesity. Smoking was defined as more than 100 cigarettes in a lifetime and alcohol consumption was defined as more than 12 drinks per year in a lifetime. Micro- albuminuria was defined as 30–300 mg/g, and macro-albuminuria was defined as ≥300 mg/g. The eGFR was calculated utilizing the CKD-EPI formula (20).

### Statistical analysis

In the descriptive analysis, continuous variables following a normal distribution are presented as the mean plus or minus the standard deviation (Mean ± SD). Those with skewed distributions are represented by the median and the interquartile range, while categorical variables are displayed as percentages. Weighted t tests or weighted chi-square tests were used to assess differences between participants grouped by grip strength tertiles. For data with skewed distribution, natural logarithmic transformation was used in the analysis. Multiple linear regression analysis was employed to examine the link between grip strength and ACR. Three distinct analytical models were constructed: Model 1 was unadjusted; Model 2 accounted for adjustments based on sex, age, and race; and Model 3 included adjustments for all the aforementioned covariates.

To enhance the robustness of our analysis, we conducted a sensitivity study using grip strength tertiles. The median value of each category of the independent variable was included in the model as a continuous variable to identify linear trends. Subsequently, subgroup analyses were performed. *p* values below 0.05 were considered statistically significant. To minimize potential bias and variation in the dataset, a weighting mechanism was used. All statistical analyses and visual representations were performed using R (version 4.2.0) and EmpowerStats (version 4.0).

## Results

### Subject characteristics

The demographic and main clinical characteristics of the study population were as follows. The mean age was 47.13 ± 16.85 years. There were 48.71% males and 51.29% females. A total of 8.07% of the participants were Mexican American, 5.79% were other Hispanic, 67.15% were non-Hispanic white, 11.28% were non-Hispanic black, and 7.70% were from other racial groups. 8.30% of the subjects had micro- albuminuria and 1.33% had macro- albuminuria.

Differences in the three grip strength classification groups were found in age, gender, race, education, alcohol consumption, smoking, hypertension, hyperlipidemia, diabetes, albuminuria, BMI, HbA1c, TC, LDL-C, HDL-C, TG, eGFR, Cr and UA (*p* < 0.05). Subjects with higher grip strength were generally younger and male. They were more likely to be of Mexican American or Non-Hispanic Black and to have higher education levels. These individuals often were smokers and drinkers, with higher BMI, eGFR, Cr, UA, TG, and LDL-C levels. They also had lower HbA1c, TC, and HDL-C levels. Additionally, these subjects were less likely to have diabetes mellitus, hypertension, or hyperlipidemia, and were less prone to albuminuria. These observations are detailed in Table 1.

**Table 1:** Study population characteristics by grip strength tertiles.

			Grip strength tertiles		
	Total	Tertiles1	Tertiles2	Tertiles3	*p-*value
Grip strength range	14.8–169.6	14.8–58.1	58.2–80.3	80.4–169.6	
N	9,638	3,199	3,222	3,217	
Age (years)	47.13 ± 16.85	53.86 ± 17.94	45.72 ± 16.37	42.69 ± 14.37	<0.0001
Gender (%)					<0.0001
Male	48.71	7.30	34.26	96.65	
Female	51.29	92.70	65.74	3.35	
Race/Ethnicity (%)					<0.0001
Mexican American	8.07	7.80	7.90	8.45	
Other Hispanic	5.79	6.83	5.26	5.40	
Non-Hispanic White	67.15	68.31	65.64	67.52	
Non-Hispanic Black	11.28	8.12	13.37	12.10	
Other Race	7.70	8.95	7.83	6.53	
Education level (%)					<0.0001
Less than 9th grade	4.53	5.82	4.51	3.47	
9-11th grade	10.66	12.20	9.28	10.58	
High school graduate	21.01	21.79	18.53	22.56	
Some college or Associate of Arts degree	32.97	32.36	33.86	32.41	
College graduate or above	30.93	27.83	33.82	30.98	
Smokers (%)					<0.0001
Yes	43.66	39.36	42.45	48.39	
No	56.34	60.64	57.55	51.61	
Drinkers (%)					<0.0001
Yes	79.25	68.57	78.73	88.56	
No	20.75	31.43	21.27	11.44	
Diabetes (%)					<0.0001
Yes	12.68	16.97	11.02	10.54	
No	87.32	83.03	88.98	89.46	
Hypertension (%)					<0.0001
Yes	32.62	39.83	29.81	29.04	
No	67.38	60.17	70.19	70.96	
Hyperlipidemia (%)					<0.0001
Yes	34.70	40.91	31.02	32.73	
No	65.30	59.09	68.98	67.27	
BMI	28.95 ± 6.84	28.29 ± 7.26	29.23 ± 7.08	29.26 ± 6.19	<0.0001
HbA1c (%)	5.62 ± 0.95	5.74 ± 1.07	5.57 ± 0.88	5.58 ± 0.89	<0.0001
UA (umol/L)	321.54 ± 83.42	293.80 ± 82.40	304.87 ± 78.01	359.29 ± 74.56	<0.0001
TC (mmol/L)	4.99 ± 1.07	5.08 ± 1.12	4.95 ± 1.08	4.94 ± 1.03	<0.0001
Cr (umol/L)	78.60 ± 26.81	72.34 ± 35.85	75.02 ± 23.25	86.96 ± 17.11	<0.0001
eGFR (mL/min/1.73 m2)	94.13 ± 22.07	90.20 ± 25.16	95.87 ± 22.04	95.84 ± 18.67	<0.0001
HDL-C (mmol/L)	1.37 ± 0.41	1.50 ± 0.41	1.42 ± 0.43	1.23 ± 0.34	<0.0001
TG (mmol/L)	1.12(0.78–1.66)	1.12(0.78–1.60)	1.06(0.74–1.57)	1.16(0.81–1.83)	<0.0001
LDL-C (mmol/L)	2.94 ± 0.91	2.93 ± 0.91	2.89 ± 0.92	3.00 ± 0.89	0.0044
Urine albumin (%)					<0.0001
Normal	90.37	85.30	91.07	94.03	
Microalbuminuria	8.30	12.54	7.74	5.21	
Macroalbuminuria	1.33	2.16	1.19	0.76	

Mean ± SD or median (interquartile range) for continuous variables and as frequencies (percentages) for categorical variables, *p*-value was calculated by weighted linear regression model or weighted chi-square test.

BMI, body mass index; HbA1c, Hemoglobin A1c; UA, uric acid; TC, total cholesterol; HDL-C, high-density lipoprotein cholesterol; TG, triglyceride; LDL-C, low-density lipoprotein cholesterol; Cr, creatinine; eGFR, estimated glomerular filtration rate.

### Association between grip strength and ACR

Our results showed a negative correlation between grip strength and ACR (Table 2). Multiple regression analyses showed that this association was significant in unadjusted model 1 [−0.77 (95% CI: −1.35, −0.20)] and in model 2 [−2.42 (95% CI: −3.38, −1.46)] adjusted for sex, age, and race. In the fully adjusted model, ACR decreased by 0.49 (95% CI: −0.93, −0.04) for each unit increase in grip strength.

**Table 2:** Association between grip strength and ACR.

	Model 1 β (95% CI) *p-*value	Model 2 β (95% CI) *p-*value	Model 3 β (95% CI) *p-*value
Grip strength	−0.77 (−1.35, −0.20) 0.0084	−2.42 (−3.38, −1.46) <0.0001	−0.49 (−0.93, −0.04) 0.0319
Tertiles 1	Reference	Reference	Reference
Tertiles 2	−37.20 (−69.87, −4.53) 0.0257	−62.61 (−0.32, 0.01) 0.0006	−14.01 (−29.89, 1.87) 0.0838
Tertiles 3	−40.96 (−72.75, −9.16) 0.0116	−120.39 (−172.01, −68.77) <0.0001	−29.22 (−52.47, −5.98) 0.0160
*P* for trend	0.0135	<0.0001	0.0136

The analytical models were defined as follows Model 1: unadjusted; Model 2: adjusted for sex, age, and race; Model 3: adjusted for age, gender, race, education, alcohol Use, smoking, hypertension, hyperlipidemia, diabetes, BMI, HbA1c, Cr, TC, TG, LDL-C, HDL-C, eGFR, and UA.

In the sensitivity analysis, where grip strength was categorized into tertiles, the fully adjusted model demonstrated a statistically significant downward trend. Specifically, the adjusted β decreased by 29.22 in the highest tertile compared to the lowest. This trend was consistent across all categories, with a *p*-value for trend less than 0.05, indicating statistical significance.

**Figure 2:**
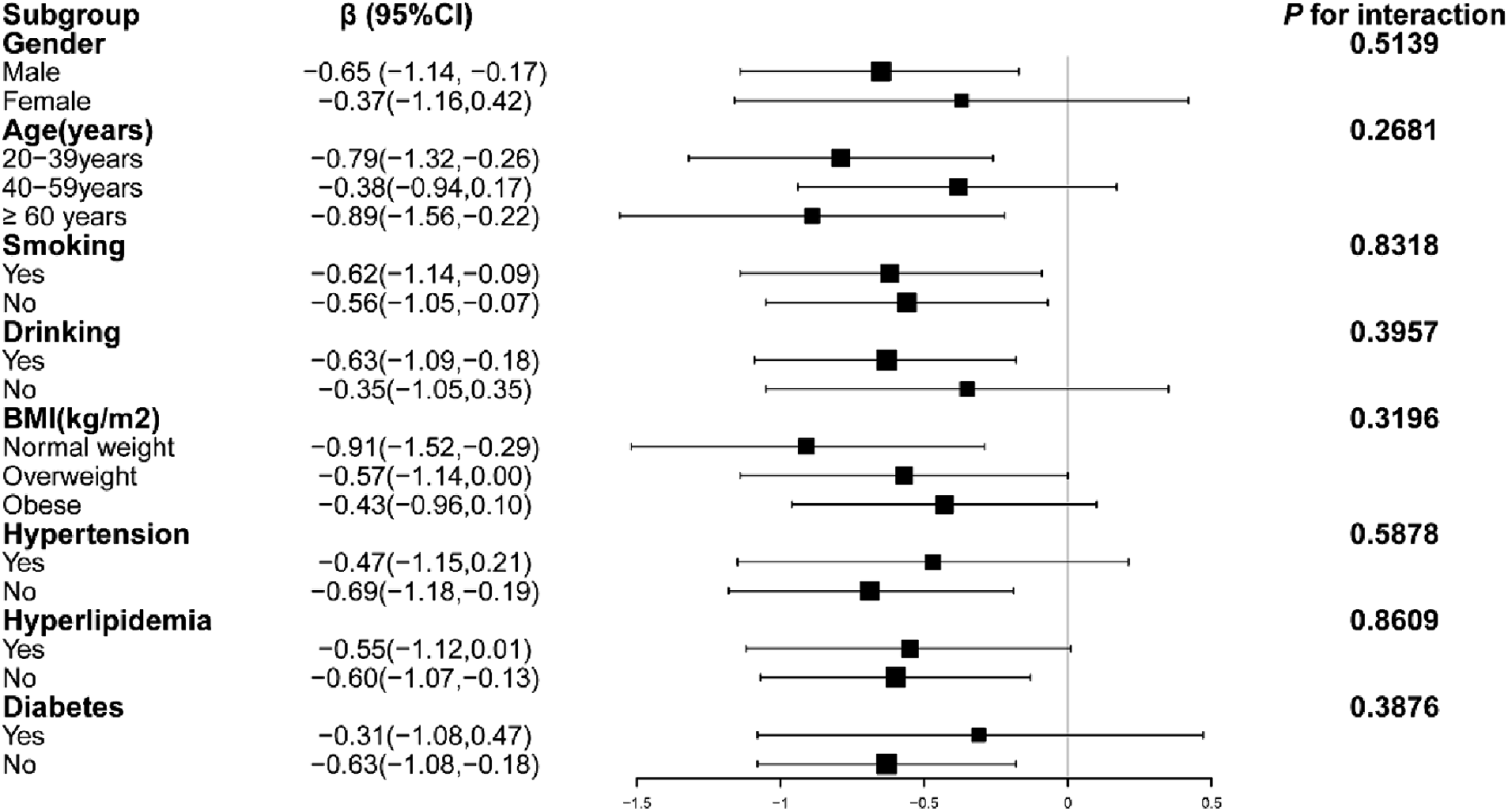
Subgroup analysis of the correlation between grip strength and ACR. Models adjusted for age, gender, race, education, alcohol Use, smoking, hypertension, hyperlipidemia, diabetes, BMI, HbA1c, Cr, TC, TG, LDL-C, HDL-C, eGFR and UA.

Figure 2 illustrates that in the subgroup analysis, factors such as age, gender, hypertension, hyperlipidemia, diabetes, smoking, alcohol consumption, and body mass index did not significantly alter the relationship between grip strength and ACR (*P* for interaction > 0.05).

## Discussion

In this study of the general U.S. population, we observed a negative association between grip strength and ACR. After controlling for confounders, each 1 kg increase in grip strength was associated with a 0.49 mg/g decrease in ACR. Subgroup analyses and interaction tests revealed that this relationship was consistent across different demographics.

This study is notably the first in the U.S. to explore the connection between grip strength and albuminuria. It builds upon findings from two prior studies in Korean populations. In a cross-sectional study conducted from 2009 to 2011, it was observed that proteinuria levels were significantly higher in the sarcopenia group compared to the non-sarcopenia group. After adjusting for confounding factors, the odds ratio for proteinuria was 2.84 in the sarcopenia group without chronic kidney disease (CKD), 3.70 in the non-sarcopenia CKD group, and 5.19 in the sarcopenia CKD group. Sarcopenia was found to elevate the likelihood of proteinuria among older individuals without CKD, and this association persisted even after controlling for factors such as obesity, hypertension, diabetes, and metabolic syndrome (21). Another study confirmed similar results, showing that this correlation exists only in the older population and loses significance in the younger population. Moreover, sarcopenia and obesity have a synergistic effect on increasing the risk of albuminuria (22). Contrastingly, a different observational cohort study, which included 892 CKD outpatient participants, yielded divergent results. This research identified a link between the stages of chronic kidney disease (CKD) and sarcopenia, but it did not find a substantial connection between proteinuria and muscle loss (23).

In these earlier studies, sarcopenia was determined based on appendicular skeletal muscle mass (ASM), contrasting with our study’s focus on muscle function as measured by grip strength. Our findings highlight a distinct and independent negative correlation between grip strength and albuminuria in the general U.S. population, consistent across gender, age, and BMI categories.

The mechanisms explaining the association between grip strength and albuminuria are unclear, and one of the possible correlates is insulin resistance. Insulin resistance is associated with episodes and severity of albuminuria, even in healthy populations, suggesting that insulin resistance itself may contribute to albuminuria (24). Research by Welsh et al. points to insulin receptors and the Akt (the v-akt murine thymoma viral oncogene homologue 1 protein) and MAPK (mitogen-activated protein kinase) signaling pathways as significant players in this process (25). The negative correlation between grip strength and insulin resistance has been demonstrated in several previous studies and was again demonstrated by measurements of grip strength and insulin resistance in adolescents in the NHANES 2011–2014 cycle (26–29). Increasing muscle strength levels through resistance training induces improvements in glucose homeostasis, reduces HbA1c levels and upregulates key proteins in the insulin signaling cascade response (30). This means that albuminuria may be reduced or prevented by improving grip strength.

Another plausible mechanism involves inflammation. Inflammation and endothelial dysfunction are markers and contributors to the development of diabetic proteinuria (31). In turn, the occurrence of proteinuria triggers renal tubular cells to secrete an abundance of chemokines, vasoactive mediators, and adhesion molecules. This secretion leads to interstitial infiltration and the activation of mononuclear inflammatory cells, ultimately resulting in additional tubular injury and enhanced interstitial fibrosis (32). Reducing inflammation levels is therefore a key target for improving albuminuria and halting the progression of kidney disease. The negative correlation between grip strength and inflammation levels has been confirmed by several studies (33). The possible mechanism is that in the presence of chronic inflammation, muscle tissue releases factors called “myokines” that reduce the synthesis of pro-inflammatory cytokines and up-regulate anti-cytokines (34). Resistance training is effective in reducing plasma pro-inflammatory factors such as C-reactive protein (35). Therefore, it is expected that resistance training will increase muscle strength and reduce inflammation levels, thus lowering urinary protein levels.

In this cross-sectional study, we identified a significant negative correlation between grip strength and albuminuria, offering a new perspective on integrated interventions to reduce albuminuria. Despite the limitations inherent to the cross-sectional design that prevent us from establishing causality, our findings align with previous studies conducted across various populations, underscoring the potential significance of muscle function for cardiac and renal health.

Given the intricate connection between albuminuria and the progression of cardiac and renal failure, targeted comprehensive interventions are of paramount importance. These interventions include dietary adjustments, pharmacological therapies, and lifestyle modifications, all aimed at reducing albuminuria and thereby diminishing the risk of cardiac and renal complications. Importantly, the safety and efficacy of low-protein diets have been validated, with no detrimental effects on muscle atrophy, muscle mass, or overall health. Moreover, the judicious application of medications such as angiotensin-converting enzyme inhibitors (ACEIs), angiotensin II receptor blockers (ARBs), sodium-glucose cotransporter 2 inhibitors (SGLT2is), glucagon-like peptide-1 receptor agonists (GLP-1 RAs), and mineralocorticoid receptor antagonists (MRAs) can further reduce albuminuria, enhance muscle strength, and improve the overall health condition (36–39).

In addition, increased physical activity is associated with a reduction in albuminuria, as shown in studies such as Böhm M’s study and the Research on Exercise Therapy for Chronic Kidney Disease (RENEXC) trial (40–42). These findings highlight the possibility that enhancing muscle strength could decrease albuminuria, thereby slowing the progression of cardiac and renal diseases.

### Study strengths and limitations

The study had several strengths: the sample size was large, appropriate adjustments were made for possible covariates, and subgroup analyses were done to enhance the reliability of the findings.

Limitations: The cross-sectional nature of the study emphasizes correlation but does not establish causation; further prospective studies are needed to confirm causation. Secondly, due to lack of drug information, we did not adequately consider the effect of certain drugs such as renin-angiotensin system inhibitors on albuminuria.

## Conclusion

In summary, our study indicates a negative association between grip strength and albuminuria within the US population. It is important to note, however, that these findings do not imply causality. To establish a definitive causal link, further comprehensive prospective research is required. Nonetheless, our results suggest that grip strength could be a valuable biomarker for the early detection of albuminuria and may assist in guiding its treatment.

## Data availability statement

The original contributions presented in the study are included in the article/supplementary materials, further inquiries can be directed to the corresponding author.

## Ethics statement

The research involving human participants underwent a thorough review and received approval from the Research Ethics Review Board of the NCHS. All patients or participants gave their written informed consent to be part of this study.

## Author contributions

LY: Writing – original draft. XH: Writing – original draft. SW: Writing – review & editing. LC: Writing – review & editing. SZ: Writing – original draft.
